# Is COVID-19 a high risk factor for lung cancer? A protocol for systematic review and meta-analysis

**DOI:** 10.1097/MD.0000000000023877

**Published:** 2021-01-08

**Authors:** Mingxin Wu, Ruiyu Mou, Xiaodi Liu, Shanqi Guo, Fanming Kong, Xiaojiang Li, Yingjie Jia

**Affiliations:** a First Teaching Hospital of Tianjin University of Traditional Chinese Medicine; b National Clinical Research Center for Chinese Medicine Acupuncture and Moxibustion, Tianjin, China.

**Keywords:** COVID-19, lung cancer, meta-analysis, protocol, risk factor, systematic review

## Abstract

**Introduction:**

COVID-19 has become a common threat to global human health and is caused by severe acute respiratory syndrome coronavirus 2 (SARS-CoV-2). Some asymptomatic patients with early-stage lung cancer who have COVID-19 receive surgical treatment but develop severe pneumonia and other complications or even experience postoperative death, and they may have a worse prognosis compared with healthy individuals infected with COVID-19. However, there is no evidence that COVID-19 is a risk factor for lung cancer patients. This systematic review aims to evaluate the incidence and prognosis of COVID-19 in lung cancer patients and provide evidence-based medical support for clinical treatment.

**Methods:**

We will search 6 medical databases to identify eligible studies published from the establishment of the database to the present. The quality of the included literature will be evaluated using the bias risk assessment tool in Cochrane 5.1.0, and a meta-analysis will be performed using Stata 14.0. Heterogeneity will be statistically assessed using χ^2^ tests.

**Results:**

The study will integrate existing research findings to investigate the prevalence and severity rate of patients with lung cancer infected with SARS-CoV-2 and analyze the prognosis and adverse clinical outcomes in patients with or without COVID-19.

**Conclusion:**

The results of this study provide evidence to support whether COVID-19 is a risk factor for lung cancer and provide guidance for clinical prevention and treatment based on the evidence obtained in light of the unpredictable threat posed by COVID-19.

**Ethics and dissemination:**

Ethics approval is not required for this systematic review as it will involve the collection and analysis of secondary data. The results of the review will be reported in international peer-reviewed journals.

**PRORPERO registration number:**

CRD42020195967.

## 1 Introduction

COVID-19 was designated as a global health emergency by the World Health Organization on January 30, 2020 and is caused by severe acute respiratory syndrome coronavirus 2 (SARS-COV-2).^[1–3]^ According to data from the Johns Hopkins Coronavirus Resource Center, as of September 15, 2020, SARS-CoV-2 has caused 29,182,198 infections and 927,015 deaths worldwide.^[4]^ The early symptoms of COVID-19 are mainly include fever, cough and fatigue. Some patients mainly exhibit digestive tract symptoms, such as vomiting and diarrhea. One week later, patients develop chest tightness, dyspnea, and respiratory distress; some patients progressed rapidly to acute respiratory distress syndrome (ARDS) and septic shock and even death.^[5]^ A retrospective study on critical COVID-19 patients showed that 67.3% of patients developed ARDS, 28.9% had acute kidney injury, 23.1% had heart injury, and 28.9% had abnormal liver function. In addition, the mortality rate was as high as 61.5% on the 28th day.^[6]^ This novel coronavirus could be detected in human respiratory epithelial cells within 96 hours in vitro. Respiratory droplets and close contact are the main routes of transmission.^[7]^

The viral genome has 5 essential genes: ribonucleoprotein (N), viral envelope (E), matrix protein (M), and spike protein (S) and RNA-dependent RNA polymerase (RdRp). Ribonucleoprotein (N) wraps the RNA genome to form a nucleocapsid surrounded by a viral envelope (E). The virus envelope contains proteins, such as matrix protein (M) and spike protein (S). Spike proteins enter cells by binding to angiotensin converting enzyme 2 (ACE-2). When cultured in vitro, the novel coronavirus can be found in human respiratory tract cells in approximately 96 hours. Virus is also found intracellularly and requires approximately 4 to 6 days for isolation and culture in Vero E6 and Huh-7 cell lines.

According to the latest data from 2019, the number of new cases of lung cancer worldwide in 2017 had increased to 2,163,130 cases, which represents a two-fold increase compared with that reported in 1990. From 1990 to 2017, the number of deaths caused by lung cancer and the disability-adjusted life years (DALY) increased by 82.30% and 61.27%, respectively. The number of new lung cancer cases and deaths and DALYs were increasing worldwide.^[8]^

Treatment for lung cancer patients simultaneously infected with COVID-19 represents a special challenge for oncologists. In this situation, clinicians must deal with the threat of 2 potentially serious health diseases. The need to isolate COVID-19 patients is inconsistent with cancer treatments. On the other hand, the relative immunosuppression risk associated with cancer treatment may also result in complications, such as pulmonary inflammation and effusion, which may make these patients at an increased risk of contracting COVID-19.^[9]^ Some lung cancer patients must delay surgery and treatment plans.^[10]^ Defects in lung structure caused by lung surgery may also make patients susceptible to infection. Airway and lung tissue anatomy changes lead to changes in the tumor environment, including increases in macrophages, immune cells and inflammatory infiltrates, which increase the risk of cytokine release.^[11]^ COVID-19 is associated with a high burden of severity in lung cancer patients, and some early lung cancer patients could also be asymptomatic for COVID-19 at the time of surgical treatment, thus increasing the risk for serious pneumonia, other complications, and postoperative death.^[12]^ In 2 lung patients who were retrospectively found to have had COVID-19, pathologic examinations revealed that apart from the tumors, the lungs of both patients exhibited patchy inflammatory cellular infiltration, but both patients did not exhibit symptoms of pneumonia at the time of operation.^[13]^ A large proportion of lung cancer patients need glucocorticoids to prevent and control cancer-related symptoms. Steroids reduce inflammation and immune cell activity, may be negatively impact COVID-19 treatment, and mask some early symptoms of SARS-COV-2 infection.^[11]^

Since the outbreak of COVID-19, a large number of studies have reported the clinical, radiological, and virologic characteristics of the disease as well as the risk factors for serious disease and death, including the age of diagnosis, complications of SARS-CoV-2 infection, ICU (Intensive Care Unit) admission rate, adverse reactions caused by CT, high SOFA score, and mechanical ventilation requirements. There was consensus that during the COVID-19 pandemic, it is appropriate to defer enrollment in lung cancer screening due to the added risks from potential exposure.^[14]^ If COVID-19 is a high risk factor for lung cancer, the routine treatment of patients with cancer has been affected during the COVID-19 pandemic, thereby indicating a poor prognosis.^[15]^ Treatment paradigms must continue to be individualized with careful consideration of risks and benefits of continuing or altering lung cancer-directed therapy.^[16]^ There are insufficient studies to accurately describe the characteristics of SARS-CoV-2 infections in patients with lung cancer. It is possible that this study will provide more evidence on these patients and SARS-CoV-2. These data may suggest the need for proactive strategies to reduce the likelihood of infection and improve early identification in this vulnerable patient population.^[17]^ Given that patients with lung cancer are generally more vulnerable to infections, systematic analysis of diverse cohorts of patients with lung cancer affected by COVID-19 is needed.^[18]^

## 2 Methods

### 2.1 Protocol and registration

We completed the systematic review protocol agreement in the international prospective register of systematic reviews (PROSPERO, https://www.crd.york.ac.uk/PROSPERO); its unique registration number is CRD42020195967.

### 2.2 Eligibility criteria

#### 2.2.1 Types of study

The studies included cohort studies, case-control studies, cross-sectional studies, and randomized controlled trials. No limitations were placed on the language or publication date. The following publications were excluded: studies using the same or overlapping data, reviews or meta-analyses, letters to editors, comments, editorials, case reports, and studies that do not include useful data and the residual effects of cross-sectional studies.

#### 2.2.2 Type of participant

All patients should be diagnosed based on the following conditions:^[19]^

1. Suspected cases: These patients exhibit an epidemiological history combined with clinical manifestations. Any one of the 2 factors should be evident, and 2 clinical manifestations should be noted. If there is no clear epidemiological history, any 2 of the clinical manifestations should be evident, and the patient is positive for new Type I coronavirus-specific IgM antibody. Alternatively, 3 of clinical manifestations are noted. a. Epidemiological history: Travel or residence history in the community where the case was reported within 14 days prior to onset of illness; patients with novel coronavirus infection within 14 days prior to onset of illness or asymptomatic or infected person has a history of exposure; exposure to fever in the community with reported cases within 14 days before the onset of the disease or patients with respiratory symptoms; cluster of disease (within 2 weeks in a small area such as home, office, school, including 2 or more cases of fever and/or respiratory symptoms in class or other places). b. Clinical manifestations: COVID-19-related clinical manifestations, such as fever and/or respiratory symptoms; exhibiting the above COVID-19 imaging characteristics; the total number of white blood cells was normal or decreased at the early stage of the disease; and the lymphocyte count was normal or decreased.^[19]^
2. Confirmed cases: Suspected cases with one of the following etiological or serological findings: a. Novel coronavirus nucleic acid was detected by RT-PCR; b. Viral gene sequencing results were highly homologous with novel coronavirus; c. Patients was positive for novel coronavirus-specific IgM and IgG antibodies; d. Novel coronavirus specific IgG antibody result changes from negative to positive or IgG antibody levels in the convalescent stage exhibit a 4-fold increase compared with that in the acute stage.^[20,21]^

There is no limit to the sample size based on gender and age.

#### 2.2.3 Type of intervention

There is no time limit, and the following comparisons will be made:

1. Comparison of infection and noninfection in lung cancer patients;
2. Comparison of severe cases of infection between lung cancer patients and nonlung cancer patients;
3. Infection in lung cancer patients and mortality in nonlung cancer patients;
4. Comparison of quality of life (QoL) between infected lung cancer patients and nonlung cancer patients;
5. Infection rate of lung cancer patients and cure rate of nonlung cancer patients.

#### 2.2.4 Outcome indicators

##### 2.2.4.1 Key outcome measures

The primary outcomes of the study include the following 2 aspects. The first key outcome measure is the prevalence of lung cancer patients diagnosed with COVID-19. The second aspect is to study the differences in the clinical outcomes of COVID-19 patients with or without lung cancer.

##### 2.2.4.2 Secondary outcomes

The secondary outcomes of the study are mainly to explore the lung cancer-specific transition to severe disease rate, response rate, QoL, and mortality of patients diagnosed with COVID-19.

##### 2.2.4.3 Patient and public involvement

No patients are involved. There are no plans to disseminate the results to study participants. The risk factors were not assessed by patients themselves in this study.

### 2.3 Retrieval strategy

Study retrieval was conducted using CNKI, WanFang, SinoMed, PubMed, EMBase, Cochrane Central Register of Controlled Trials, and Web of Science databases, and the retrieval time included all studies published to date. In addition, references can be traced, and other resources, such as conferences and books, can be used to supplement relevant literature. See Annex 1 for details.

### 2.4 Literature screening

Two researchers independently conducted literature screening and data analysis, and the retrieval results were imported into Endnote X9. Duplicate articles will be deleted. Articles that do not meet the standards will also be deleted. Any differences will be discussed and resolved with the assistance of a third party. The included studies will be evaluated by the Jadad scale and the Cochrane systematic evaluation method.

### 2.5 Data extraction

The studies retrieved during the searches will be screened for relevance, and only those studies meeting the standards will be included. After the studies are filtered, we will extract data from most studies. Two reviewers will independently extract the following information:

1. General information (title, first author, and publication date).
2. Baseline data (type of study, sample size, duration of intervention, therapeutic scheme, follow-up time, and main variables).
3. Basic characteristics of participants (authors, year of publication, study location, age, and patient characteristics).
4. Results (primary endpoint, secondary endpoint, and adverse events).

Two reviewers will be responsible for extracting and managing the data, which will be input into an EXCEL spreadsheet, and discrepancies will be clarified with the help of a third researcher.

### 2.6 Methodological quality assessment

We will independently assess any bias in the included studies according to the criteria from the Cochrane Handbook, version 5.3.0, which assesses random sequence generation, allocation concealment, blinding of participants and personnel, blinding of outcome assessment, incomplete outcome data, selective reporting, and other sources of bias.^[22]^ Studies are classified as high quality, low quality and unclear. If information on randomization, blindness, outcome assessment methods, outcome data and other potential bias is insufficient to make a risk judgment, this study will still be included in the systematic review, and the risk of selection bias will be assessed as ambiguous. The overall risk of bias is estimated to be low only if all areas are rated as low risk of bias; otherwise, the overall assessment of bias in the study is considered high risk. We will use the bias risk graph to summarize the results of the bias risk assessment. Any disagreement will be resolved by discussion or by reference to a third investigator.

### 2.7 Data analysis

Stata 13.0 was used to draw the network diagram of the intervention measures in the included study. Revman 5.3 was used for direct meta-analysis. Given that the outcome indicators included counting data and that odds ratio (OR) and confidence interval (95% CI) were used as effect size, heterogeneity was analyzed in combination with I2. Here, *I*^2^ ≥ 50% or *P* < .1 indicates a high risk of heterogeneity, and the source of heterogeneity will be further analyzed. If heterogeneity cannot be explained by subgroup analysis, meta-regression, or sensitivity analysis, then the random effect model will be selected to combine the effect size. GeMTC 0.14.3 was used for Bayesian mesh meta-analysis, and the effect index of each intervention was calculated to predict the possible optimal ranking.

## 3 Discussion

In the age of COVID-19, the best management of lung cancer patients remains unknown, and the oncology community should raise awareness to prevent an increase in cancer-related and infectious mortality. While it may seem reasonable to suspend or delay cancer treatment in some circumstances, the risks/benefits and end results of these biases remain to be measured. A new Global Registry is attempting to gather global data to develop a tailored risk assessment strategy for lung cancer patients. Despite the current lack of reliable data, it is important to establish an international consensus on COVID-19 testing in lung cancer patients, where early detection of COVID-19 may lead to targeted treatment.

At least 100,000 surgical procedures have been delayed in Canada since early March due to COVID-19. This study reviewed the data of 1524 cancer patients, and the COVID-19 infection rates in cancer patients was 0.79% (12/1524 cases 95% CI: 0.3%–1.2%), which is higher than the cumulative incidence of all confirmed COVID-19 cases reported in Wuhan in the same period (0.37%; 41081152/11000 cases on February 17, 2020). Cancer patients from epidemic centers exhibit an increased risk of SARS-COV-2 infection compared to the community (OR, 2.31; 95% CI, 1.89–3.02), but less than half of these patients are receiving aggressive antitumor treatment. In addition, the findings suggest that hospitalization and repeated hospitalization are potential risk factors for COVID-19 infection. Positive measures should be taken to reduce the frequency of hospitalization of cancer patients during an epidemic.

ASCO confirmed that the fatality rate of cancer patients infected with COVID-19 was 7.6%, whereas the total mortality rate of COVID-19 patients was 3.8%. In patients with complications, including cardiovascular disease, diabetes, high blood pressure, and chronic respiratory system disease, the mortality rates were 13.2%, 9.2%, 8.4%, and 8.0%, respectively. Liang provides the most detailed data in Lancet Oncol. The prospective cohort of 1571 COVID-19 patients finds that patients with a history of cancer exhibited an increased incidence of serious events (percentage of patients requiring invasive ventilation, ICU admission, or death) in cancer patients compared to other patients. According to this report, the 18 patients represent a heterogeneous group and are not an ideal representation of all cancer patients.

The Chinese Center for Disease Control and Prevention reported 72,314 cases (including confirmed cases, suspected cases and clinically diagnosed cases) and describe the epidemiological characteristics and analysis of COVID-19 patients with underlying diseases. A total of 107 cases (0.5%) cancer patients were included in the study, and 6 cases of death were noted. The mortality rate was 5.6%, which is greater than that noted in the overall population with a crude mortality of 2.3%.^[10]^

These findings suggest that during the COVID-19 epidemic, cancer patients may be vulnerable and exhibit a poor prognosis based on the following reasons. Given that cancer patients need to go to the hospital repeatedly, the COVID-19 exposure risk is significantly increased in cancer patients compared with the general population. Cancer patients are relatively older, and the ability to prevent infection is poor in this population. After surgery, chemoradiotherapy or immunotherapy, cancer patients exhibit impaired immune function. Thus, these patients exhibit poor disease resistance, and the risk of infection is high.^[13]^ Cancer patients are relatively old and exhibit poor physical performance. These patients often exhibit comorbidities.

Clinical decisions should be made individually by taking into account factors, such as the risk of cancer recurrence when treatment is delayed, modified, or interrupted and the number of treatment cycles completed. Some issues still need to be clarified. For example, is it reasonable to delay surgery and neoadjuvant therapy? Are the potential hazards that may result from delayed or interrupted treatment inconsistent with the prevention and treatment of COVID-19 infection? Whether prophylactic growth factors used in chemotherapy regimens and prophylactic antibiotics valuable in maintaining patient health and making them less susceptible to COVID-19 complications? Do antiviral drugs have therapeutic effects on suspected or confirmed tumors in infected patients?

In summary, cancer patients, especially lung cancer patients, infected with COVID-19 exhibit severe symptoms and a high mortality rate. Thus, this patient population is a focus of attention for epidemic prevention. Patients receiving antitumor therapy for lung cancer with fever and respiratory symptoms need to be differentiated. For lung cancer patients, refined and individualized management is needed during the COVID-19 epidemic to maximize benefits.[[] Xu Y, Liu HS, Hu K, et al. Clinical Management of Lung Cancer patients during the Outbreak of 2019 Novel Coronavirus Disease (COVID-19).^[23]^

## Author contributions

**Data curation:** Ruiyu Mou, Shanqi Guo.

**Formal analysis:** Mingxin Wu, Xiaodi Liu.

**Funding acquisition:** Xiaojiang Li, Yingjie Jia.

**Investigation:** Fanming Kong, Yingjie Jia.

**Methodology:** Fanming Kong, Shanqi Guo.

**Project administration:** Ruiyu Mou.

**Resources:** Xiaojiang Li, Yingjie Jia.

**Software:** Ruiyu Mou, Xiaodi Liu.

**Writing – original draft:** Mingxin Wu, Xiaodi Liu.

**Writing – review & editing:** Mingxin Wu, Shanqi Guo.
